# Reply to Kersting et al. Comment on “Wunsch et al. The Impact of COVID-19 on the Interrelation of Physical Activity, Screen Time and Health-Related Quality of Life in Children and Adolescents in Germany: Results of the Motorik-Modul Study. *Children* 2021, *8*, 98”

**DOI:** 10.3390/children8070533

**Published:** 2021-06-23

**Authors:** Kathrin Wunsch, Carina Nigg, Claudia Niessner, Steffen C. E. Schmidt, Doris Oriwol, Anke Hanssen-Doose, Alexander Burchartz, Ana Eichsteller, Simon Kolb, Annette Worth, Alexander Woll

**Affiliations:** 1 Institute of Sports and Sports Science, Karlsruhe Institute of Technology, 76131 Karlsruhe, Germany; carina.nigg@partner.kit.edu (C.N.); claudia.niessner@kit.edu (C.N.); Steffen.schmidt@kit.edu (S.C.E.S.); doris.oriwol@partner.kit.edu (D.O.); alexander.burchartz@kit.edu (A.B.); ana.eichsteller@kit.edu (A.E.); simon.kolb@kit.edu (S.K.); alexander.woll@kit.edu (A.W.); 2 Institute of Movement and Sport, University of Education Karlsruhe, 76133 Karlsruhe, Germany; anke.hanssen-doose@ph-karlsruhe.de (A.H.-D.); annette.worth@ph-karlsruhe.de (A.W.)

In reply to the comment of Kersting et al. [1], there is a need to mention that there are multiple health behaviors, which may relate to health-related quality of life. Nudelman and Shiloh attempted to cluster these behaviors and created a taxonomy consisting of 66 health behaviors [2]. In a network analytic approach, they screened all those behaviors for interrelatedness and centrality [3] and found eating behavior to be one of the most central ones. This, in turn, may substantiate the assumption determined by the authors of the respective comment that dietary behavior may be as important in the prediction of HRQoL as physical activity and screen time. A recent review and meta-analysis of Wu and colleagues [4] support this assumption, as the authors found that dietary behavior was indeed associated with health-related quality of life in children and adolescents.

However, next to nutrition, Nudelman and Shiloh [2] identified three other relevant domains of health behaviors: general well-being, risk avoidance, and health maintenance. Our current examination focused on the latter domain, which includes physical (in-) activity and exercise. Considering those four domains, we agree that nutrition might be an important factor when investigating relationships between health behaviors and health-related quality of life. For a comprehensive investigation, we would like to add that health behaviors of the remaining two categories, risk avoidance (e.g., substance use) and general well-being including stress (e.g., stress, relationships), should also be added.

Furthermore, we recommend that the directionality of the associations still needs to be investigated. Contrary to our hypothesis, we did not find physical activity to be a significant predictor of health-related quality of life during the COVID-19 pandemic. Considering the nutrition domain, there is only a limited number of studies that investigated longitudinal associations between dietary behaviors and health-related quality of life in children and adolescents [4], warranting future research to investigate if a positive longitudinal relationship exists across the developmental period of childhood and adolescence. In the context of the COVID-19 pandemic, only a few cross-sectional investigations exist, examining changes in nutrition behavior in the pediatric population [5], while we are not aware of any studies that investigated associations between nutrition and health-related quality of life.

In conclusion, we would recommend investigating associations between health behaviors and health-related quality of life beyond physical activity and screen time, including nutrition and dietary behaviors, in addition to health behaviors concerning general well-being and risk avoidance.

## Data Availability

Not appplicable.
